# Relationship between circulating VCAM-1, ICAM-1, E-selectin and MMP9 and the extent of coronary lesions

**DOI:** 10.6061/clinics/2018/e203

**Published:** 2018-05-19

**Authors:** Jéssica Cavalcante dos Santos, Marina Sampaio Cruz, Raul Hernandes Bortolin, Katiene Macêdo de Oliveira, Jéssica Nayara Góes de Araújo, Victor Hugo Rezende Duarte, Ananília Medeiros Gomes da Silva, Isabelle Cristina Clemente dos Santos, Juliana Marinho de Oliveira Dantas, Maria Sanali Moura de Oliveira Paiva, Adriana Augusto Rezende, Mario Hiroyuki Hirata, Rosario Dominguez Crespo Hirata, André Ducati Luchessi, Vivian Nogueira Silbiger

**Affiliations:** I Departamento de Analises Clinicas e Toxicologicas, Universidade Federal do Rio Grande do Norte, Natal, RN, BR; II Departamento de Cardiologia, Hospital Universitario Onofre Lopes, Universidade Federal do Rio Grande do Norte, Natal, RN, BR; III Departamento de Analises Clinicas e Toxicologicas, Faculdade de Ciencias Farmaceuticas, Universidade de Sao Paulo, Sao Paulo, SP, BR

**Keywords:** VCAM-1, Cardiovascular Diseases, Atherosclerosis, Inflammatory Molecules

## Abstract

**OBJECTIVES:**

Inflammatory molecules play a role in the development of atherosclerosis, which is the primary origin of cardiovascular disorders. However, to the best of our knowledge, no study has attempted to investigate the relationship between these circulating molecules and the prediction of cardiovascular risk. The present study aimed to investigate the relationships of vascular cell adhesion molecule-1, intercellular adhesion molecule-1, E-selectin and matrix metalloproteinase 9 serum concentrations with the extent of coronary lesions.

**METHODS:**

Seventy-four individuals who were undergoing coronary angiography for the first time for diagnostic purposes were enrolled in this study. The extent of the coronary lesion was assessed using the Friesinger Index, and subjects were classified into four groups: no lesions, minor lesions, intermediate lesions and major lesions. Serum biochemical parameters and serum concentrations of vascular cell adhesion molecule-1, intercellular adhesion molecule-1, E-selectin and matrix metalloproteinase 9 were analyzed.

**RESULTS:**

The vascular cell adhesion molecule-1 concentration was higher than 876 ng/mL in individuals with intermediate and major lesions (*p*<0.001 and *p*=0.020, respectively). Moreover, logistic regression analysis showed that these patients had an increased risk of having an intermediate lesion (*p*=0.007). Interestingly, all individuals with major lesions had vascular cell adhesion molecule-1 concentrations higher than 876 ng/mL. No association was found between the concentrations of the other proteins and the Friesinger Index.

**CONCLUSIONS:**

Serum vascular cell adhesion molecule-1 may be associated with the extent of coronary lesions. Moreover, it may represent an alternative to improve the cardiovascular risk classification in patients without acute coronary syndrome.

## INTRODUCTION

Cardiovascular disease is the leading cause of death worldwide. Obtaining knowledge about its physiopathology seems to be the best approach to reducing morbidity, mortality, and health care costs, since there are no non-invasive methods, laboratory tests or clinical symptoms that are capable of detecting which individuals will develop coronary artery disease (CAD) 1.

Atherosclerosis, the primary origin of most common cardiovascular disorders, is an inflammatory process with slow and progressive development. This process involves increased expression of cellular adhesion molecules and enhanced cell migration, low-density lipoprotein (LDL) recruitment and fibrous tissue proliferation that lead to partial or total blood flow obstruction 2-4. During this process, migrating cells secrete more inflammatory cytokines and matrix metalloproteinases, leading to plaque rupture 5 and thrombus formation 4. The detection of atherosclerotic plaques at this inflammatory stage could allow the prevention of future acute cardiovascular events 6.

Studies have shown that the determination of soluble adhesion molecules, such as intercellular adhesion molecule-1 (ICAM-1), vascular cell adhesion molecule-1 (VCAM-1) and E-selectin, can reflect the inflammatory process in the endothelium. Together, these molecules form an integrated and overlapping system for the transport of leukocytes into the vascular wall and play an active role in the development of atherosclerotic plaques 7. Moreover, matrix metalloproteinases (MMPs) play important roles in thrombogenesis by disrupting atherosclerotic plaques and participating in the degradation of the extracellular matrix 8,9.

However, few studies have shown an association between increased expression of these proteins in tissues and their soluble level in the plasma. Also, to the best of our knowledge, no studies have associated these molecules with the early atherosclerotic process and the coronary atherosclerotic burden in humans 2,10,11. Previous studies have investigated these molecules in patients with acute coronary syndrome and even post-infarction patients, but no study has evaluated these molecules prior to cardiovascular events.

Therefore, the aim of this study was to investigate a possible relationship between circulating VCAM-1, ICAM-1, E-selectin or MMP9 and the extent of coronary lesions in patients without acute coronary syndrome nor post-infarction patients. These results may contribute to the identification of potential biomarkers and the development of non-invasive methods for the diagnosis of atherosclerosis, thus reducing health costs and cardiovascular risk.

## MATERIALS AND METHODS

### Study population

Seventy-four male and female individuals aged between 30 to 74 years who were undergoing coronary angiography at the hemodynamics Department of University Hospital Onofre Lopes (HUOL) for diagnostic purposes were enrolled in the present study. Subjects with cardiomyopathy, including Chagas disease, heart valve disease, congenital diseases, and pericarditis, and subjects with an angioplasty or stent were excluded from this study. Additionally, patients with familial hypercholesterolemia or the presence of chronic kidney disease, liver failure, endocrine disorders (except for diabetes mellitus), inflammatory diseases, malignant diseases, blood disorders, autoimmune diseases were excluded. The study was conducted in accordance with the guidelines set by the Ethics in Research Committee of the Federal University of Rio Grande do Norte and complies with the Declaration of Helsinki (protocol CAAE 0001.0.051.294-11). All participants signed an informed consent form.

### Extent of coronary lesions

The extent of the coronary lesions was assessed using the Friesinger Index 12–14. Each of the three main coronary arteries (anterior descending, circumflex and right coronary) was scored separately from zero to five. The subjects were classified as follows: no lesion when the Friesinger Index was equal to 0; minor lesion when the Index varied from 1–5; intermediate lesion when the Index varied from 6–10; and major lesion when the Index varied from 11–15, as adapted from Chagas et al. 15.

### Blood samples and biochemical analysis

Peripheral venous blood samples were obtained from the patients. Fasting serum glucose, total cholesterol and fractions, urea, creatinine K, uric acid, alanine aminotransferase (ALT) and aspartate aminotransferase (AST) were measured using colorimetric and colorimetric-enzymatic (Labtest, Minas Gerais, Brazil) methods on a semi-automatic biochemical analyzer (BIO-2000 IL, Bioplus, SP, Brazil). The LDL level was calculated according to the formula described by Friedewald et al. 16.

A multiplex immunoassay using polystyrene beads based on the Luminex technology was performed since the VCAM-1, ICAM-1, E-selectin and MMP9 molecules were found in small amounts in the blood. The Millipex^®^ MAP Human CVD Panel 1 Immunoassay Kit (Merck KGaA, Darmstadt, Germany) was used according to the manufacturer's instructions. Briefly, the samples were mixed with antibody-linked polystyrene beads in 96-well filter-bottom plates and incubated at room temperature for 2h, followed by overnight incubation at 4°C. The plates were vacuum filtered, washed twice with wash buffer, and then incubated with a biotinylated detection antibody for 1h at room temperature. Streptavidin–phycoerythrin was added to the sample. After incubation for 30 min at room temperature and two additional vacuum washes, the samples were resuspended in reading buffer. Each sample was measured in duplicate. The plates were read using a Luminex^®^ 100/200™ System (Luminex, Austin, TX, USA) with a lower bound of 100 beads per sample per cytokine.

### Statistical analysis

The statistical analyses were performed using the SPSS software v20.0 (SPSS Inc., Chicago, IL, USA). The data were categorized in two conditions: 1. Friesinger Index groups (no lesion, minor lesion, intermediate lesion and major lesion) and 2. Tertile concentrations of inflammatory molecules (1^st^, 2^nd^ and 3^rd^). Categorical variables were shown as percentages and compared using the Chi-square test. Continuous variables were presented as the mean and standard deviation and compared using the Kruskal–Wallis test or ANOVA, followed by Tukey's post hoc test according to the normality of the distribution assessed using the Kolmogorov-Smirnov test. Spearman's correlation was used to calculate correlations between the inflammatory protein concentrations and the Friesinger Index. Logistic regression analyses were used to evaluate the relationships between the increases in serum inflammatory molecules and the Friesinger Index. The level of statistical significance was set as *p*<0.05.

## RESULTS

The patients were classified into four groups according to the extent of the coronary lesions evaluated by the Friesinger Index as follows: no lesion (31.0%), minor lesion (29.8%), intermediate lesion (27.0%) and major lesion (12.2%). The demographic, anthropometric, clinical and biochemical data collected from these groups are shown in Table 1 and Table 2. Significant differences in the clinical and biochemical data were not found among the groups.

**Figure 1:**
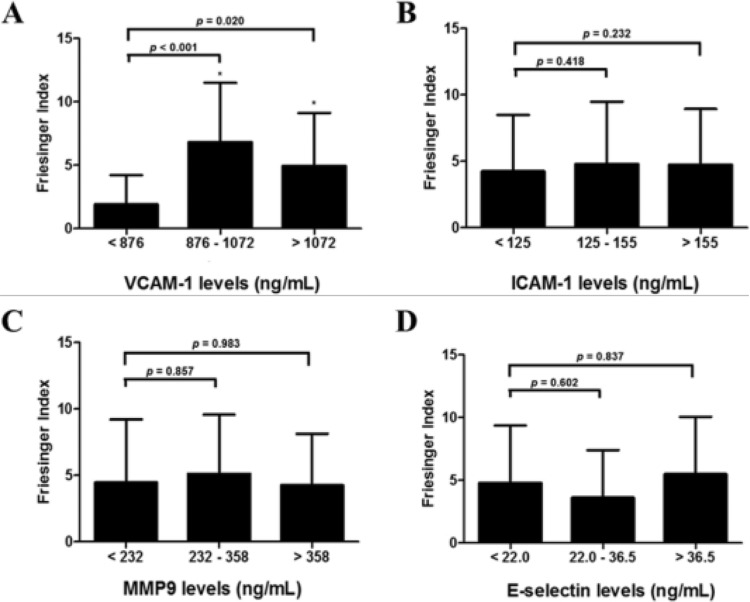
Friesinger Index in patients undergoing coronary angiography for the first time stratified by tertiles of serum protein levels. (A) Individuals with higher VCAM-1 concentrations (≥876 ng/mL) are likely to have higher Friesinger Index values. Serum (B) ICAM-1, (C) MMP9 and (D) E-selectin levels were not associated with increased Friesinger Index values. Data are shown as the mean ± SD. ^*^ represents a significant difference compared with the 1^st^ tertile.

The categorization of the adhesion molecules and MMP9 into tertiles showed that patients with intermediate and major lesions had VCAM-1 concentrations >876 ng/mL (2^nd^ and 3^rd^ tertiles), whereas patients with no or minor lesions had VCAM-1 concentrations <876 ng/mL (1^st^ tertile, *p*<0.05, Figure 1).

Additionally, the patients with VCAM-1 concentrations >876 ng/mL had a 9.81-fold higher risk of having intermediate lesions (*p*<0.01; OR: 9.81; 95% CI: 1.84 – 52.38, Table 3). In this study, all patients with major lesions had VCAM-1 concentrations >876 ng/mL. E-selectin, ICAM-1 and MMP9 were not associated with the Friesinger Index, although VCAM-1 was correlated with ICAM-1 and the extent of the coronary lesion (*p*<0.01, r^2^=0.40; and *p*=0.02, r^2^=0.29, respectively).

## DISCUSSSION

The primary origin of CAD is atherosclerosis, which is a chronic inflammatory process that induces the expression of adhesion molecules at the endothelial membrane. VCAM-1, ICAM-1 and E-selectin promote the connection of leukocytes and their migration into the intima, whereas MMP9, a metalloproteinase, plays a special role in the instability of atherosclerotic plaques. These molecules can also be found in the serum in a soluble form due to the proteolytic rupture of cellular membranes in which adhesion molecules are expressed. Therefore, their measurement can be evaluated in several clinical situations in which inflammation is a causal contributor, making these molecules easily accessible serum markers 2,17,18.

Interestingly, in the present study, it was observed that patients with intermediate and major lesions had high VCAM-1 concentrations (>876 ng/mL). In addition, the logistic regression analysis indicated that patients subjected to coronary angiography with VCAM-1 levels higher than 876 ng/mL had a 9.8-fold greater risk of having atherosclerotic lesions (intermediate lesions), whereas ICAM-1 showed no correlation with the Friesinger Index. All patients with major lesions had a VCAM-1 concentration >876 ng/mL, thereby suggesting a possible VCAM-1 cut-off value that may aid in the determination of the extent of coronary lesions.

A previous study with 855 patients diagnosed with stable carotid atherosclerosis over a 6.2-year period observed that VCAM-1 values higher than 837 ng/mL increased the cardiovascular mortality risk by 2.5-fold and predicted future fatal cardiovascular events, whereas ICAM-1 values higher than 335 ng/mL increased that risk by 3.4-fold; these results indicated that these molecules were strong and independent predictors of mortality in patients with stable carotid atherosclerosis 2. In contrast, another study that enrolled 15 patients with a high cardiovascular risk during stent implantation found that the VCAM-1 serum concentrations were not different from the basal levels (prior to stent implantation) and were independent of the ICAM-1 serum concentration 19. Despite these controversial results, to the best our knowledge, no study has attempted to evaluate the association of serum VCAM-1 with cardiovascular risk in patients without acute coronary syndrome, thus supporting the needed for new studies.

No significant difference was observed between serum levels of ICAM-1 and the extent of coronary lesions; however, an indirect positive correlation between these factors was revealed, as VCAM-1 correlated with the Friesinger Index and VCAM-1 correlated with ICAM-1 serum levels. This result could be due to the lower ICAM-1 concentrations found in the subjects enrolled in the present study (<250 ng/mL). Moreover, the considerable controversies were found in the literature about the predictive values of ICAM-1 and VCAM-1 may be explained by the characteristics of both molecules. ICAM-1 seems to be a natural marker of pro-inflammatory conditions, whereas VCAM-1 is rapidly induced by pro-atherosclerotic circumstances.

Regarding MMP9 and E-selectin, significant results were not found in the present study. MMP9 plays a critical role in the later stages of atherosclerotic lesions, particularly in the rupture of the atherosclerotic plaque 17,20. MMP9 concentrations were significantly increased in aortic samples from 89 patients who underwent a surgical procedure for abdominal aortic aneurysms 21. Also, soluble MMP9 was associated with vulnerable atherosclerotic plaques in 88 patients who experienced a major adverse cardiovascular event in a cohort study over a 3-year period 20. In the present study, only patients with no history of acute coronary syndrome who underwent coronary angiography for the first time were included, and we found that 60.8% of our patients had only minor or no atherosclerotic lesions. These aspects may account for the absence of high serum levels of MMP9 in our study population.

Regarding E-selectin, the absence of an association between E-selectin and the extent of coronary lesions in the present study could be explained by the involvement of this molecule in the early nonspecific stages of leukocyte adherence and rolling. This result was also observed by other authors, whom also did not associate soluble E-selectin with adverse clinical outcomes in 855 patients diagnosed with stable carotid atherosclerosis 2 or in a study evaluating 65 patients with acute coronary syndrome 22.

The present results are important, particularly regarding VCAM-1 serum levels, because no atherosclerotic lesions were detected in 31% of the patients, and the high frequency of patients without lesions who were subjected to an invasive procedure in both studies indicates a clear need to establish new tests to complement and aid CAD diagnosis. Similar data were recently reported in a study of 100 patients of both genders undergoing coronary angiography for diagnostic purposes, showing that 37% of those patients had no atherosclerotic lesions 12. Our hypothesis is also supported by other authors, whom have emphasized the absence of non-invasive methods, clinical symptoms or laboratory tests that are capable of detecting individuals who will develop CAD 1.

A major limitation of the present study was the small sample size, as our pilot study population consisted of patients without previous acute cardiac events who were referred for diagnostic coronary angiography for the first time. However, it is important to highlight that this was the first study to evaluate circulating inflammatory molecules and their relationships with the extent of coronary lesions in patients without acute coronary syndrome.

Some of the established major vascular risk factors are not observed in over 20% of patients who develop coronary events 23. Thus, using markers involved in the atherosclerotic process may lead to better, earlier and more accurate risk predictions for CVDs. In addition, non-invasive methods and laboratory tests play an important role in decreasing health costs and improving quality of life among patients.

In conclusion, circulating VCAM-1 might be associated with the extent of coronary lesions in patients with a risk of developing acute coronary syndrome. In addition, this result suggests that VCAM-1 may also be associated with the prediction of cardiovascular disease and the severity of atherosclerosis. Further investigation is required to validate this hypothesis and to determine whether monitoring VCAM-1 serum levels may be valuable for the assessment of cardiovascular risk.

## AUTHOR CONTRIBUTIONS

Silbiger VN and Luchessi AD were responsible for the original concept of the study and study design. Santos JC, Oliveira KM, Araújo JN, Duarte VH, Silva AM and Santos IC were responsible for the recruitment of the patients, collection of the samples and the laboratory analysis. Bortolin RH, Santos JC, Cruz MS, Dantas JM, Paiva MS, Luchessi AD and Silbiger VN interpreted the results and performed the statistical analyses. Bortolin RH, Santos JC, Cruz MS, Araújo JN, Luchessi AD and Silbiger VN wrote the manuscript. Bortolin RH, Rezende AA, Hirata MH, Hirata RD, Luchessi AD and Silbiger VN were responsible for the critical revision of the manuscript.

## Figures and Tables

**Table 1:** Demographic, anthropometric and clinical data from patients classified according to the extent of the coronary lesion.

Variable	Total (74)	No lesion (23)	Minor lesion (22)	Intermediate lesion (20)	Major lesion (9)	*p-value*
Age, years	60±10	56±9	61±10	63±10	62±7	0.061
Gender, male	55.4 (41)	43.5 (10)	59.1 (13)	70.0 (14)	44.4 (4)	0.307
BMI, kg/m^2^	26.5±5.4	27.4±4.3	25.5±7.6	26.4±4.2	26.3±3.4	0.752
Obesity, %	17.6 (13)	26.1 (6)	18.2 (4)	15.0 (3)	0.0 (0)	0.437
Dyslipidemia, %	85.1 (63)	69.6 (16)	90.9 (20)	95.0 (19)	100 (9)	0.085
Diabetes, %	32.4 (24)	34.8 (8)	22.7 (5)	35.0 (7)	44.4 (4)	0.647
Hypertension, %	81.1 (60)	78.3 (18)	81.8 (18)	80.0 (16)	88.9 (8)	0.919
Diastolic pressure, mmHg	84±18	80±9	83±22	87±17	89±29	0.588
Systolic pressure, mmHg	143±26	137±23	143±24	151±34	141±20	0.472
Treated hypertension, %	77.0 (57)	78.3 (18)	81.8 (18)	70.0 (14)	77.8 (7)	0.715
Sedentary lifestyle, %	55.4 (41)	52.2 (12)	50.0 (11)	60.0 (12)	66.7 (6)	0.804
Alcoholism, %	16.2 (12)	21.7 (5)	18.2 (4)	5.0 (1)	22.2 (2)	0.445
Smoking, %	23.0 (17)	21.7 (5)	18.2 (4)	25.0 (5)	33.3 (3)	0.826
AIM family history, %	43.2 (32)	39.1 (9)	31.8 (7)	50.0 (10)	66.7 (6)	0.294

Data are shown as the mean ± SD or the percentage for categorical variables (number of patients) and compared using the Kruskal–Wallis test or ANOVA for continuous variables and the Chi-square test for categorical variables. BMI: body mass index; and AMI: acute myocardial infarction. *p*-values <0.05 were considered statistically significant.

**Table 2:** Biochemical data according to the extent of the coronary lesion.

Variable	Total (74)	No lesion (23)	Minor lesion (22)	Intermediate lesion (20)	Major lesion (9)	*p-value*
Glucose, mmol/L	6.17±2.95	5.76±2.34	5.68±1.82	7.10±4.37	6.38±2.62	0.408
Total cholesterol, mmol/L	4.73±1.20	4.47±1.04	4.58±1.02	5+11±1.44	4.91±1.42	0.305
HDL cholesterol, mmol/L	0.98±0.27	1.04±0.31	0.93±0.22	0.93±0.26	1.03±0.29	0.414
LDL cholesterol, mmol/L	2.86±1.07	2.64±0.90	2.68±0.89	3.16±1.31	3.22±1.28	0.261
Triglycerides, mmol/L	1.97±1.48	1.70±1.30	2.11±1.44	2.37±1.87	1.46±0.76	0.157
ALT, µKat/L	0.51±0.36	0.50±0.39	0.52±0.23	0.48±0.45	0.58±0.39	0.298
AST, µKat/L	0.57±0.32	0.65±0.38	0.54±0.29	0.51±0.22	0.63±0.39	0.639
Urea, mmol/L	13.45±3.72	13.03±3.68	13.52±3.26	14.10±4.40	12.98±3.82	0.864
Creatinine K, µmol/L	84.0±258	85.0±24.2	81.6±20.0	89.7±28.3	74.6±36.7	0.505
Uric acid, mmol/L	0.29±0.09	0.29±0.10	0.30±0.10	0.28±0.10	0.28±0.08	0.965
E-selectin, ng/mL	32.5±16.8	33.5±19.8	30.4±14.6	34.4±17.3	30.6±14.6	0.855
VCAM-1, ng/mL	990.0±270.3	994.3±330.3	899.7±214.7	1080.2±282.0	998.9±118.3	0.196
ICAM-1, ng/mL	152.2±53.7	152.6±68.4	161.9±58.0	144.9±39.5	144.0±21.1	0.695
MMP9, ng/mL	324.2±156.4	348.8±198.5	302.9±127.7	351.8±146.3	252.2±102.6	0.325

Number of individuals in parentheses. Continuous variables are presented as the mean ± standard deviation and compared using the Kruskal–Wallis test or ANOVA. Categorical variables were compared using the Chi-square test. HDL: high-density lipoprotein; LDL: low-density lipoprotein; AST: alanine aminotransferase; and ALT: aspartate aminotransferase. *p*-values <0.05 were considered statistically significant.

**Table 3:** Logistic regression analysis: influence of increased VCAM-1 concentrations (>876 ng/mL) on the extent of the coronary lesion.

Dependent variable	Covariable	OR	(95% CI)	*p*-value
VCAM-1 >876 ng/mL	No lesion	1		
	Minor lesion	1.309	0.405-4.226	0.652
	Intermediate lesion	9.818	1.840-52.384	**0.007**

Logistic regression analysis was performed to determine the Friesinger Index using a VCAM-1 serum concentration >876 ng/mL as a dependent variable. This analysis was not performed with the Major lesion group because, in this study, all patients with major lesions had VCAM-1 concentrations >876 ng/mL.

OR: odds ratio; and CI: confidence interval.
